# Using High-Density SNP Array to Reveal Selection Signatures Related to Prolificacy in Chinese and Kazakhstan Sheep Breeds

**DOI:** 10.3390/ani10091633

**Published:** 2020-09-11

**Authors:** Yi Wang, Zhigang Niu, Zhengcheng Zeng, Yao Jiang, Yifan Jiang, Yugong Ding, Sen Tang, Hongcai Shi, Xiangdong Ding

**Affiliations:** 1 Laboratory of Animal Genetics, Breeding and Reproduction, Ministry of Agriculture, National Engineering Laboratory for Animal Breeding, College of Animal Science and Technology, China Agricultural University, Beijing 100193, China; wangyi_chin@163.com (Y.W.); 996845493@cau.edu.cn (Z.Z.); jiangyao133996@126.com (Y.J.); jiangyf2020_2020@163.com (Y.J.); 2 Key Lab of Reproduction & Breeding Biotechnology of Grass Feeding Livestock of MOA, P.R.C. Xinjiang Academy of Animal Science, Urumqi 830000, China; xjnzg@126.com (Z.N.); skdown@foxmail.com (Y.D.); tangsen@xjaas.net (S.T.)

**Keywords:** selection signatures, sheep, litter size, SNP

## Abstract

**Simple Summary:**

Genetic improvement of litter size trait in domestic animals is an appealing way to improve production efficiency. In our study, the selection signatures between multiparous and uniparous sheep populations are identified, so that potential pathways and candidate genes related to litter size were screened out. Our findings help better understand the mechanisms of selection underlying the prolificacy trait in sheep and other mammals.

**Abstract:**

Selection signature provides an efficient tool to explore genes related to traits of interest. In this study, 176 ewes from one Chinese uniparous breed and three Kazakhstan multiparous breeds are genotyped using Affymetrix 600K HD single nucleotide polymorphism (SNP) arrays, F-statistics (Fst), and a Cross Population Extend Haplotype Homozygosity Test (XPEHH). These are conducted to identify genomic regions that might be under selection in three population pairs comprised the one multiparous breed and the uniparous breed. A total of 177 and 3072 common selective signatures were identified by Fst and XPEHH test, respectively. Nearly half of the common signatures detected by Fst were also captured by XPEHH test. In addition, 1337 positive and 1735 common negative signatures were observed by XPEHH in three Kazakhstan multiparous breeds. In total, 242 and 798 genes were identified in selective regions and positive selective regions identified by Fst and XPEHH, respectively. These genes were further clustered in 50 gene ontology (GO) functional terms and 66 Kyoto Encyclopedia of Genes and Genomes (KEGG) pathways in enrichment analysis. The GO terms and pathways were relevant with reproductive processes, e.g., oxytocin signaling pathway, thyroid hormone synthesis and GnRH signaling pathway, vascular smooth muscle contraction and lipid metabolism (alpha-Linolenic acid metabolism and Linoleic acid metabolism), etc. Based on the findings, six potential candidate genes *ESR1*, *OXTR*, *MAPK1*, *RYR1*, *PDIA4*, and *CYP19A1*, under positive selection related to characteristics of multiparous sheep breeds were revealed. Our results improve our understanding of the mechanisms of selection that underlies the prolificacy trait in sheep, and provide essential references for future sheep breeding.

## 1. Introduction

From the point of view of population genetics, when a novel mutation is subjected to the selection pressure over a long time, it will generate “selection signature”, demonstrating some distinguished features on the genome, e.g., unusual linkage disequilibrium (LD) and changed population frequency [1]. Therefore, identifying the selection signatures underlying phenotypic difference can contribute to target causal variants for breeding, as well as explore the mechanisms of evolution. Furthermore, it can also help us to reveal the genetic basis of complex traits with phenotypic difference [2,3]. Li et al. (2019) [4] detected strong signatures of selection in genes associated with the local adaptation of Tibetan sheep; Yuan et al. (2019) [5] genotyped 78 meat Lacaune and 103 milk Lacaune sheep to identify the selection signature related to ovine milk traits. Many methods have been proposed to detect pre-mentioned selection signatures, including Fst test based on population differentiation [6], the integrated Haplotype Homozygosity Score (iHS) [7] and the Cross Population Extend Haplotype Homozygosity Test (XPEHH) [8] based on linkage disequilibrium, etc. These methods have been widely applied in many studies to identify selection signatures. In this study, we employed the Fst and XPEHH test to detect the selection signatures between populations. 

Litter size is one of the most important reproductive traits in sheep, as well as in other domestic animals, and has always been regarded as a critical index affecting the reproductive performance and productivity. Sheep presents variable litter size within and among breeds attribute to the natural and artificial selection for higher prolificacy during the long breeding history. For instance, Booroola Merino is well known as its reproductive characteristic with two offspring [9], and the Chinese local breeds, Hu sheep and small tail Han sheep are also characterized by high-prolificacy trait [10]. Many studies reported a series of genes or causal mutations associated with litter size through different genetic analyses in sheep [11]. Galloway et al. (2000) [12] detected *FecXI* and *FecXH* mutations in gene *BMP15* associated with litter size in Romney sheep. Mulsant et al. (2001) [9] reported a mutation *FecBB* in gene *BMPR1B* in Booroola Merino associated with the highly prolific phenotype. Vage et al. (2013) [13] found that *FecGF* mutation in gene *GDF9* was related to larger litter size in Norwegian White sheep and Finn sheep through a genome-wide association study. Miao et al. (2016) [14] identified a set of differential expressed genes in different sheep breeds explaining the variation of fecundity by the integrated analysis of miRNAs and lncRNAs. Although the fecundity of sheep could be influenced by the interaction of environment (i.e., climate, nutrition, and stocking density) [15], previous researches indicated that genetic factor usually plays critical roles in the differential reproductive performance of sheep. 

**Figure A1:**
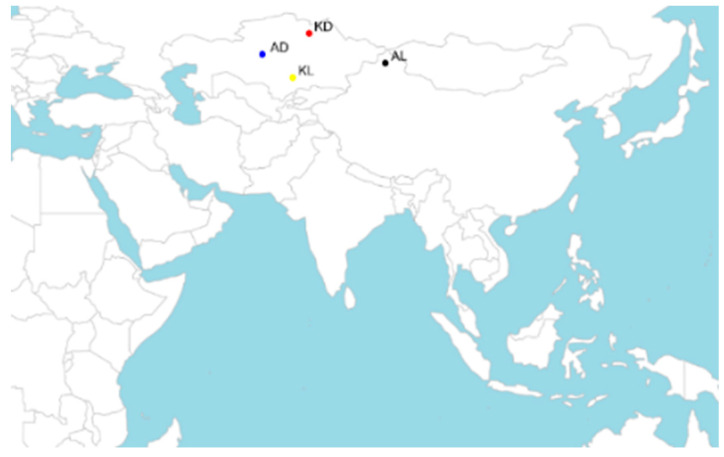
The geographical locations of four sheep breeds.

Four sheep populations (from one local Chinese breed and three local Kazakhstan breeds) were sampled, with the aim of exploring specific selection signatures for litter size in sheep. In addition, the geographic distribution of these sampled populations was shown in Figure A1. Of these, Aletai sheep, has a close relationship with Kazakstan sheep [16], mainly distributed in Xinjiang province, China, usually with one offspring [17]. Three Kazakhstan populations, Aldabas, Kurdyuchnyj and Karakul, with a high frequency of two offspring, were not only adapted to the local environment with harsh conditions but were also important genetic resources [18]. Our results will provide an essential reference for better understanding of the genetic mechanism of reproduction and further genetic improvement of litter size in sheep breeding.

## 2. Materials and Methods 

### 2.1. Samples and Phenotype

According to the farms’ reproduction records, ewes of Aletai sheep with single offspring and ewes of three Kazakhstan breeds with two offspring were collected. In total, 176 ewes were sampled in this study, including Aletai sheep (AL; *n* = 44), Aldabas (AD; *n* = 36), Kurdyuchnyj (KD; *n* = 48), and Karakul (KL; *n* = 48).

### 2.2. SNP Genotyping and Quality Control

DNA of each individual was isolated from blood, and all DNA samples were genotyped using 600 K Affymetrix Ovine HD genotyping arrays (Affymetrix, Santa Clara, CA, USA), including 633,619 SNPs across the entire sheep genome. The SNPs were re-mapped on the Ovis aries 3.1 genome assembly according to the position information provided by Affymetrix and SNPdb database. The whole procedure for collecting blood samples was carried out in strict accordance with the protocol approved by the Animal Welfare Committee of China Agricultural University (permit number DK996).

In order to improve the quality of genotyping data, genotype quality control was performed using PLINK v1.90 [19] for each group with the following criteria: (1) The individuals with >0.1 genotype missing rate were excluded; (2) SNPs with missing rate >0.1 were removed. After genotype quality control, three individuals (two from AL, one from KL) were removed, 591,189, 588,581, 570,692, and 560,856 SNPs remained for AD, KD, KL, and AL, respectively.

### 2.3. Principal Components Analysis (PCA), Population Admixture Analysis, and LD Decay

After filtering the individuals and SNPs, three methods were applied to analyze the genetic diversity among populations. Principal components analysis (PCA) was performed using the PLINK v1.90 software [19] to visualize patterns in relationships between individuals with filtered SNPs. Afterwards, population admixture analysis was performed by the ADMIXTURE program [20] to estimate the proportion of common ancestors among the four populations. Three scenarios of populations (K ranging from 2 to 4) were estimated using the cross-error estimation for genetic clustering, and the iteration times were set as 10. Additionally, levels of linkage disequilibrium (LD) for each sheep population were evaluated by genotypic correlation coefficient (*r*^2^) using PopLDdecay software [21]. The options were set as: “-MaxDist 300 kb”, and the visualization of LD decay among sheep populations across the whole genome was generated using self-written R script.

### 2.4. Identification of Selection Signatures 

To detect potential selection signatures across the genome, Fst and XPEHH tests were employed to detect the selection signatures between breeds. The approaches were proved with high power in selection signatures with approximately fixed or fixed alleles. We firstly chose the uniparous AL as the common reference, and the other three multiparous populations as observed population, respectively. For each population pair compared of AL and one multiparous population, the common SNPs were used for calculating Fst and XPEHH values. The pairs of AD vs. AL, KD vs. AL, and KL vs. AL separately has 554,521, 555,037, and 547,884 common SNPs. 

In this analysis, the single locus analysis method Fst proposed by Weir and Cockerham (1984) [6] was first employed to quantify the degree of population differentiation. The calculating of Fst was completed using VCFtools using non-window approach [22]. The Fst statistics ranges from 0 (identical population) to 1 (complete differentiation), and the top 1% SNPs were empirically considered as significant signatures in this study. Different from Fst, XPEHH is a haplotype-based method. In our study, haplotypes were firstly constructed in each breed by SHAPEIT [23], and XPEHH statistics based on the extended haplotype were calculated for each population pair using SELSCAN [24]. Since the XPEHH statistics approximately follow a normal distribution, the XPEHH values were normalized firstly. Then, the significance test of standard normal distribution (*p* < 0.05) was used to determine the variations caused by selection between populations, and the positive and negative XPEHH values represent the selection respectively occurred in the observed and reference population. Considering the selection regarding multiparous populations, the genes harbored in significant signatures with positive XPEHH values were used for further bioinformatics analysis. 

### 2.5. Functional Annotation for Selection Signatures

According to the findings of selection signature, the common signatures of three population pairs were selected, and each core SNP of the common signatures was extended 200 kb towards upstream and downstream to be defined as selective regions. Candidate genes harbored in these regions were annotated based on the NCBI database (https://www.ncbi.nlm.nih.gov/). Because the annotation of the sheep genome is incomplete, corresponding human genomic information was regarded as a reference. The human orthologous genes were generated by the program of BioMart (http://www.biomart.org/). We further performed bioinformatics analyses to explore potential biological significance of genes harbored in these selective regions. A KOBAS 3.0 [25] (http://www.kobas.cbi.pku.edu.cn/kobas3) webserver was employed to perform enrichment analyses for biological processing GO (gene ontology) terms and KEGG (Kyoto Encyclopedia of Genes and Genomes) pathways.

## 3. Results

### 3.1. PCA Analysis, STRUCTURE Analysis and LD Decay

**Figure 1:**
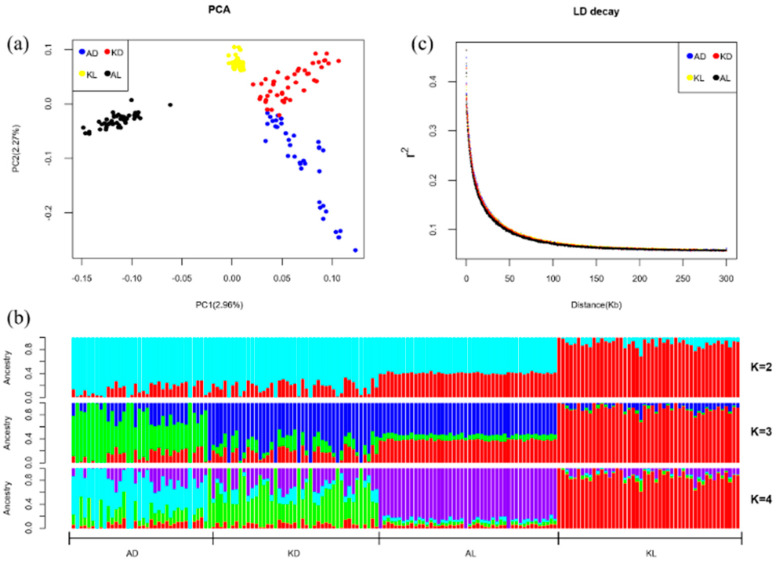
Population genetics analysis. (**a**) Principal components analysis (PCA) plot of four breeds, indicating genetic variation along the PC1 and PC2. The dots represent Aldabas (blue), Kurdyuchnyj (red), Karakul (yellow) and Aletai (black). (**b**) Population assignment proportions per individual based on results from ADMIXTURE analysis (K = 2 to K = 4). In the plot, each vertical bar represents a single individual, and the different colors reflect the genetic contribution from each of the components. (**c**) The decay of linkage disequilibrium in each breed, the *r*^2^ is the mean of the *r*^2^ in 50 kb windows.

Figure 1 shows the results of population genetics analysis among four sheep populations. The principal component analysis demonstrated the separation of individuals from a different geographic origin (Figure 1a). From the PCA diagram, individuals in the same population were clustered together. Likewise, AL was distantly related to Kazakhstan breeds, while AD, KD and KL were close but separated into different populations, due to different geographical origins. Figure 1b further illustrates the degree of separation of the four sheep populations and shows a clustering on the four populations with different values of K, the number of clusters. K values differentiate KL from the other populations (K = 2), AD from KD and AL (K = 3), and KD from AL (K = 4), indicating that AD, KD and AL are typical populations with multiple mixed ancestor sources, while KL was relatively represented by a single blood source (K = 2~4). It represents that all four populations have multiple ancestral sources, reflecting the genetic exchange among them. The influence of domestication could be reflected in the linkage disequilibrium (LD) levels in each population, showing that selection can promote the decrease of genetic diversity in the population and the enhancement of the correlation (linkage degree) between loci. Interestingly, the LD decay (Figure 1c) among four populations represented a similar trend. The averaged *r*^2^ between two SNPs with a distance of 20 kb, was 0.15 and the inflection point of LD decay was obtained at adjacent loci with a distance of 50 kb. The results suggested that the selecting pressure on the four populations were not much different, implying these populations might experience a similar process of domestication.

### 3.2. The Selection Signatures

**Figure 2:**
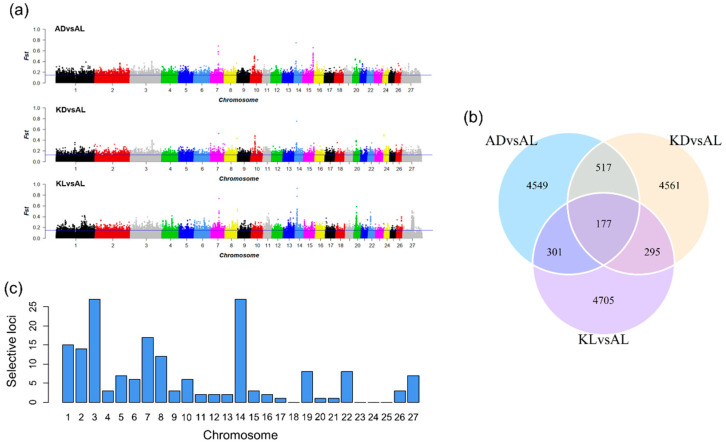
The selection signatures detected by Fst test. (**a**) Manhattan plots reflect the distribution of Fst scores between each population pairs (the top 1% suggestive line: ADvsAL, 0.15; KDvsAL, 0.13; KLvsAL, 0.15). (**b**) The venn plot indicates the counts of the top 1% signatures in each population pair and the overlap region represent the counts of common SNPs among three pairs. (**c**) The distribution of common SNPs on chromosomes. AD, Aldabas; AL, Aletai; KL, Karakul; KD, Kurdyuchnyj.

Table 1 summarizes the selective signatures observed in the three population pairs (AD vs. AL, KD vs. AL, and KL vs. AL). For the Fst test, the top 1% Fst values were set as the threshold to determine outliers, and the threshold values were 0.15, 0.13, and 0.15. Correspondingly, 5545, 5550 and 5479 SNPs were identified as outliers for each population pairs, respectively (Figure 2a). In addition, these SNPs were harbored in 1705, 1716, and 1404 selective regions detected in corresponding population pair. The venn plot (Figure 2b) indicates a total of 177 common SNPs identified as selection signatures among three population pairs. Meanwhile, a bar histogram (Figure 2c) illustrates the distribution of these common signatures on autosomes, indicating the selection mainly occurred on chromosome 1, 3, 7, and 14.

**Figure 3:**
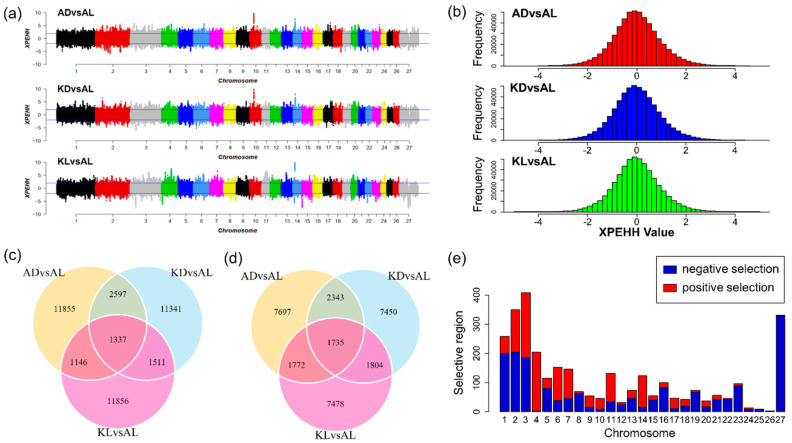
The selection signatures detected by Cross Population Extend Haplotype Homozygosity Test (XPEHH) test. (**a**) Manhattan plots reflect the genome-wide distribution of selection signatures detected by the XPEHH test in each population pair. The multiparous sheep breeds (AD, KD, and KL) are defined as observed population, respectively, and the AL sheep is the reference population. (**b**) The histogram indicates the distribution of XPEHH scores of each population pair. (**c**) The counts of the positive selection signatures in each population pair and the overlap region represent the counts of common core SNPs among three pairs. (**d**) The counts of the negative selection signatures in each population pair and the overlap region represent the counts of common core SNPs among three pairs. (**e**) The distribution of common core SNPs on chromosomes. The bars represent positive selection signatures (red) and negative selection (blue).

**Figure 4:**
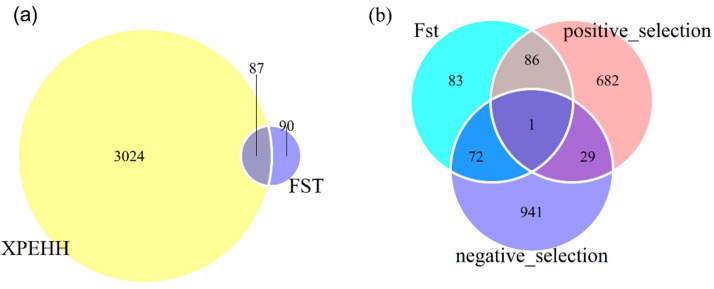
Venn plots represent: (**a**) The common selection signatures obtained by Fst and XPEHH test; (**b**) the genes under selection in three population pairs.

As shown in Figure 3b, the standardized XPEHH scores approximately followed normal distribution. Hence, outliers were identified through a normal test. The positive and negative XPEHH scores represent the selection happened in the observed and reference population, respectively. As shown in Table 1 and Figure 3a, for three population pairs, 16,935, 16,786, and 15,850 core SNPs were detected and correspondingly, 1399, 1409, and 1203 selective regions were identified in the observed populations AD, KD, and KL. Likewise, 13547, 13,332, and 12,789 core SNPs in 1416, 1471, and 1314 selection regions were detected in common reference population AL. Figure 3c,d show that 1337 common positive selection were detected in three observed populations, and 1735 common negative core SNPs were identified in AL. In total, 3111 common core SNPs were obtained in three population pairs (Figure 3c,d). Figure 3e presents the distribution of these core SNPs on chromosomes, reflecting positive selection signatures were mainly distributed on chromosome 2, 3, 4, 6, 14, and negative selection signatures were mainly observed on chromosome 1, 2, 3, 27. Particularly, chromosome 4 and 26 had positive selection signatures only, and chromosome 25 had only negative selection signatures. Additionally, nearly half of the common signatures detected by Fst were also captured by XPEHH test (Figure 4a).

### 3.3. Functional Annotation

Through identification of selective signatures between one uniparous breed and other three multiparous breeds, 242 genes were identified in selective regions of Fst test, 798 and 1043 genes were detected in positive and negative selective regions of XPEHH test. Around 66% of genes identified by Fst were included in the gene sets by XPEHH. Of the genes identified by XPEHH, 30 genes were detected in both positive and negative selective regions (Figure 4b), it will be discussed later. Finally, 953 genes harbored in selective regions identified by Fst and XPEHH (positive selective regions) were used for further biological information analyses in the GO and KEGG databases using KOBAS 3.0 webserver.

### 3.4. GO Term Enrichment Analysis

**Figure 5:**
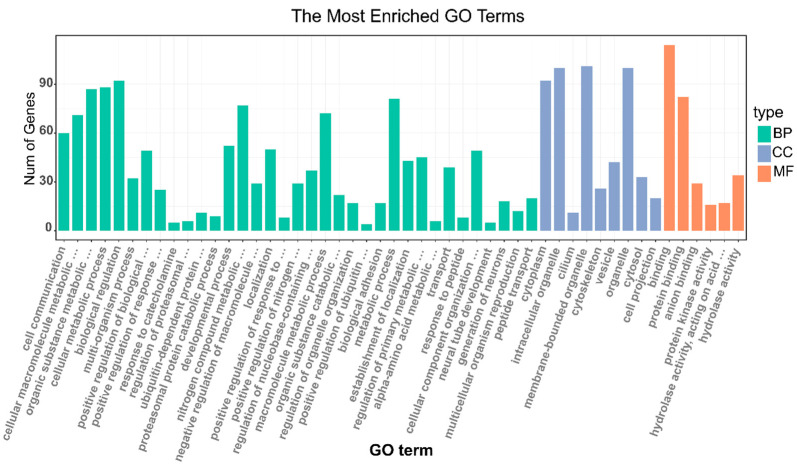
The gene ontology (GO) enrichment analysis of genes under positive selection.

According to the results of GO enrichment analyses, 953 genes under selection were further clustered in 50 GO functional terms (Figure 5, Table S1), 35 out of 50 (70%) GO terms were classified as a biological process, such as cellular metabolic process, multi-organism process and developmental process, etc. Among the biological processes, the most abundant terms were biological regulation (GO: 0065007) with 92 genes, followed by the cellular metabolic process (GO: 0044237) with 88 genes. Membrane-bounded organelle (GO: 0043227) with 101 genes was the most abundant terms in the cellular component. Binding (GO: 0005488) with 114 genes was most dominant in molecular function. Among them, one important term closely related to reproduction, the multicellular organism reproduction was enriched, it contained 12 genes, including the previously reported *ESR1*, *OXTR*, and *STAT5B* (Table 2).

### 3.5. Pathway Enrichment Analysis

**Figure 6:**
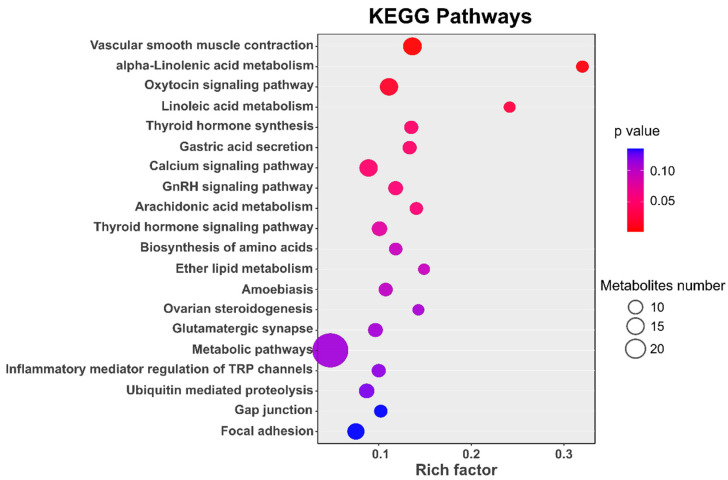
The top 20 pathways involving genes under positive selection.

KEGG enrichment analyses detected a total of 66 pathways (*p* < 0.05) relevant to genes within selective regions (Table S2) and seven pathways exhibited the strong enrichment statistical signal (corrected *p* < 0.05). Figure 6 presents the top 20 significant pathways. Of these, for the multiparous sheep, the highly represented pathways were also associated with lipid metabolism (e.g., alpha-Linolenic acid metabolism, Linoleic acid metabolism, Arachidonic acid metabolism and ether lipid metabolism) and vascular smooth muscle contraction (e.g., the Vascular smooth muscle contraction and Calcium signaling pathway) (Figure 6). Meanwhile, some known pathways related to reproduction were found to be significantly enriched. As shown in Table 2, five pathways were related to reproduction, and the genes involved in these pathways were also presented in Table 2, in all, 39 unique genes were identified relating to reproduction through these pathways.

### 3.6. Candidate Genes Related to Litter Size

Through the functional annotation analyses, genes involved in reproduction-related processes or pathway were analyzed together to find candidate genes related to high prolificacy. Six candidate genes, presented in Table 3—*OXTR* and *CYP19A1*) were identified by both Fst and XPEHH, and another four genes were detected by XPEHH. All these genes were involved in GO terms or pathway. *OXTR* and *ESR1* were presented in the reproduction-related GO term, multicellular organism reproduction. Meanwhile, *OXTR*, *MAPK1*, and *RYR1* were involved in Oxytocin signaling pathway (hsa04921), *ESR1*, *MAPK1*, and *PDIA1* in Thyroid hormone signaling pathway (hsa04919).

## 4. Discussion

In this study, genomic selection signatures related to litter size in sheep were detected using high-density SNP array in 176 sheep from one Chinese uniparous breed and three Kazakhstan multiparous breeds. The whole-genome single locus Fst statistics and haplotype-based XPEHH scores were calculated for each population pair comprising one multiparous breed and the uniparous breed. Compared to XPEHH, the selective SNPs and selective regions obtained by Fst were much less (Table 1). As the haplotype-based method, XPEHH detected the core SNPs as the representative of the corresponding fixed regions based on haplotype, it can utilize more information of LD within one region, while the single locus Fst test only calculated the diversity of two loci between populations, even using the slide-window strategy, Fst could not make full use of the SNPs in the window together, and sometimes the division of window is not reasonable, due to the disperse of LD, resulting in the lower efficiency on the detection of selection signatures of Fst. In addition, different from using the normal test to determine outliers, SNPs located at the extreme 1% of the Fst values were considered as outliers empirically, and the threshold values for all three population pairs were near 0.15 (0.15, 0.13 and 0.15). This meant only the highly differentiated SNPs could be selected, and the moderate genetic difference (Fst values ranging from 0.05 to 0.15) [26] were ignored.

As the haplotype-based approach, XPEHH can identify positive and negative selection signatures in the observed and reference populations, respectively. In this study, no overlaps were found in positive and negative selection signatures, while 30 genes were detected both in positive and negative selective regions by XPEHH. It was mainly due to the overlaps between the positive and negative selective regions after extending the selected core SNPs, e.g., ENSOARG00000004311 gene (location: chr11, 26416218bp-26416934bp) was identified by the positive selective region (location: chr11, 26354799bp-27023145bp) and the negative selective region (location: chr11, 26107221bp-26565054bp). The corresponding core SNPs were located at chr11:26554799bp and chr11:26365054bp, the close distance of core SNPs lead to the overlap of positive and negative selective regions, further resulting in the same genes identified by positive and negative selection signatures.

The enrichment analysis found that the genes under selection were involved in 60 pathways, and five out of them were related to reproduction (Table 2). For example, oxytocin signaling pathway was known as the most well-established roles in stimulating uterine contractions during parturition and lactation containing 17 genes in all. Thyroid hormone synthesis was identified since it is essential for vertebrate embryogenesis and fetal maturation. Moreover, thyroid hormones triiodothyronine (T3) and thyroxine (T4) are critical for normal development, growth and metabolic homeostasis [27]. Besides, thyroid hormone deficiency during pregnancy was reported in rat, which caused a decrease of litter size [28]. GnRH signaling pathway, and Ovarian steroidogenesis are classical signaling pathways related to follicle development. GnRH signaling pathway has been shown to regulate gonadotropin-releasing hormone (GnRH), a precondition of the subsequent hormonal cascade which could induce the ovulation [29,30]. Ovarian steroidogenesis plays a role in normal uterine function, establishment and maintenance of pregnancy. The gene members of Ovarian steroidogenesis included the reported genes associated with sheep litter size, such as hormone regulate gene *IGF1*, and the famous main role gene of multiparous sheep, oocyte-derived factor *BMP15* [31].

We also found that the genes under selection to be overrepresented in pathways related to lipids (e.g., alpha-Linolenic acid metabolism, Corrected *p* = 0.005; Linoleic acid metabolism, Corrected *p* = 0.02; Arachidonic acid metabolism, Corrected *p* = 0.05; ether lipid metabolism, *p* < 0.05), which were reported to be relevant to lipogenesis in Taihu pigs [32]. This finding indicated that the genes related to lipids traits have experienced intensive selection, and might be correlated with the fat tail trait of Aldabas and Kurdyuchnyj sheep in this study. In addition, vascular smooth muscle contraction pathway was found with the strongest enrichment statistical score (corrected *p* = 0.0027), and Calcium signaling pathway also was significantly enriched (corrected *p* < 0.05). Previous studies have reported that blood pressure is regulated by vascular smooth muscle contraction, which is triggered by an increase in intracellular free calcium concentration ([Ca^2+^]) [33]. Although the data of blood pressure in multiparous sheep have not been reported during gestation, the higher blood pressure symptom is observed in human in the twin pregnancies than singleton pregnancies [34]. Moreover, vascular remodeling in the uterine and systemic circulation is important to meet the metabolic demands of the mother and developing fetus [35]. We inferred that these pathways overrepresented in multiparous sheep breeds may relate to the psychological changing in ewes in order to meet the higher metabolic demands during the twin pregnancies.

In this study, six potential candidate genes related to litter size experienced selection signatures in all three multiparous sheep populations (Table 3). All these genes were involved in the critical pathways or GO terms of the reproductive process. For example, the *ESR1* (estrogen receptor-1) gene was found in thyroid hormone signaling pathway, which was a key gene affecting estrogen biosynthesis. Besides that, some studies reported that *ESR1* plays a critical role in follicular growth and ovulation in ewes [36] which was also an important candidate gene of litter size in sheep [37]. As for *MAPK1* (mitogen-activated protein kinase 1), it could mediates luteinizing hormone-induced breakdown of communication and oocyte maturation in rat ovarian follicles [38]. *RYR1* (ryanodine receptor) was identified taking effect on regulating calcium release in oocytes [39], which also play roles in oocyte maturation [33,38]. The difference between the *RYR1* genotypes were significant at the number of offspring in pigs [40]. Another candidate gene, *PDIA4* (protein disulfide isomerase family A, member 4), one of the redox genes whose expression patterns are related to oocyte quality [41]. Also, *PDIA4* gene was reported expressed in ovaries and associated with litter size in pigs [42]. According to our findings, *OXTR* (oxytocin receptor) and *CYP19A1,* which were identified by both Fst and XPEHH test could be the most potential genes affecting sheep litter size. Likewise, these two genes were repeatedly found in the results of enrichment analysis (Table 2). In addition, the oxytocin receptor (*OXTR*) gene is known to be important during and after the ovulatory stimulus and expressing in many tissues, including brain, thymus, ovary, and testis [43]. During reproduction, *OXTR* could bind to OXT (oxytocin), while OXT plays a role in steroidogenesis, ovulation, luteinization, and luteal regression, in the mammalian ovary [44]. Recently research suggested that OXT is associated with larger litter sizes and signals of positive selection for *OXTR* forms were found in Cebidae sheep [43]. *CYP19A1* gene in Ovarian steroidogenesis pathway encodes an estrogen-synthesizing enzyme aromatase, which is a mono-oxygenase and catalyzes many of the reactions associated with sterogenesis and the conversion of androgens to estrogen [45]. Previous studies reported that *CYP19A1* played a critical role in gonadal development in sheep, and also involved in the development of follicular follicles and follicular atresia in bovine [46,47]. In Small Tail Han, one Chinese representative multiparous sheep breed, TIAN et al. (2019) [48] reported that *CYP19A1* gene mainly expressed in ovary and hypothalamus, which suggested that *CYP19A1* gene could promote the physiological function of ovary and negatively regulates hypothalamus during estrus or ovulation.

In order to explore the genetic mechanism of litter size on a molecular level, previous studies focused on genes relevant with ovulation rate, oocyte and follicle development [49,50]. These candidate genes (*ESR1*, *OXTR*, *MAPK1*, *RYR1*, *PDIA4*, and *CYP19A1*) may be the promising resource to explore the further mechanisms of high prolificacy in sheep, as well as other mammals.

## 5. Conclusions

With the help of whole genome selective sweep analysis, we detected selection signatures among the sheep genome using the Fst and XPEHH test. Gene enrichment analyses based on selection signatures suggested that six potential candidate genes related to litter size are worthy of further functional validation to reveal the underlying mechanisms of litter size in sheep. Our results will provide an essential reference for further genetic improvement of litter size in sheep breeding.

## Figures and Tables

**Table 1:** The statistics of selection signatures and selective regions in three population pairs obtained by Fst and XPEHH.

Breed Pair ^1^	The NO. of SNP	Fst		XPEHH^+ 2^		XPEHH^− 3^	
		Selective SNPs (Threshold Value)	Regions	Core SNPs	Regions	Core SNPs	Selective Regions
**AD-AL**	542516	5545 (0.15)	1705	16,935	1399	13,547	1416
**KD-AL**	543134	5550 (0.13)	1716	16,786	1409	13,332	1471
**KL-AL**	536778	5479 (0.15)	1404	15,850	1203	12,789	1314

^1^ AD-AL represents the population pair of Aldabas and Aletai sheep, where AD and AL are observed population and reference population, respectively. KD and KL represent Kurdyuchnyj and Karakul. ^2^ Positive selection happened in the observed population. ^3^ Negative selection happened in the reference population.

**Table 2:** GO terms and pathways related to reproduction involved genes in selective regions in four sheep breeds.

Term	Database	ID	No. of Genes	*p*-Value	Corrected *p*-Value ^1^	Genes
Multicellular organism reproduction	Gene Ontology	GO: 0032504	12	0.002209	0.109913	*ESR1|OXTR|STAT5B|CATSPERG|GGN|SIRT2|* *TDRD7|MEA1|SLC26A3|SMAD5|PDGFRA|BBS4*
Oxytocin signaling pathway	KEGG PATHWAY	hsa04921	17	3.79 × 10^−5^	0.01319	*ADCY5|OXTR|MYLK|GNAS|MYLK3|RYR1|MAPK1|* *MYLK4|EEF2K|CAMK2B|PRKAG2|CALML4|* *PLA2G4E|PLA2G4D|PLA2G4F|NFATC2|PPP3R1*
Thyroid hormone synthesis	KEGG PATHWAY	hsa04918	10	0.000367	0.045264	*ADCY5|ASGR2|ASGR1|ATP1B2|GNAS|GPX8|PAX8|* *SLC26A4|PDIA4|ATP1A3*
GnRH signaling pathway	KEGG PATHWAY	hsa04912	11	0.000534	0.050691	*ADCY5|GNAS|MAPK1|PRKCD|CAMK2B|GNA11|* *EGR1|CALML4|PLA2G4E|PLA2G4D|PLA2G4F*
Thyroid hormone signaling pathway	KEGG PATHWAY	hsa04919	12	0.001109	0.077681	*MED12L|ATP1B2|TP53|KAT2A|MAPK1|ITGAV|* *PLCE1|TSC2|DIO2|ESR1|GSK3B|ATP1A3*
Ovarian steroidogenesis	KEGG PATHWAY	hsa04913	7	0.002069	0.107993	*ADCY5|GNAS|PLA2G4E|PLA2G4D|PLA2G4F|* *CYP19A1|HSD17B2*

^1^ Corrected *p*-value here is a corrected value by FDR correction method Benjamini and Hochberg.

**Table 3:** The candidate genes for litter size under selection in three multiparous sheep breeds from the critical KEGG pathways and GO terms.

Candidate Genes	GO Terms and KEGG Pathways				Test ^2^
	Term	ID	*p*-Value	Corrected *p*-Value ^1^	
*ESR1*	multicellular organism reproduction	GO: 0032504	0.002209	0.109913	XPEHH
	Thyroid hormone signaling pathway	hsa04919	0.001109	0.077681	
*OXTR*	multicellular organism reproduction	GO: 0032504	0.002209	0.109913	Fst/XPEHH
	Oxytocin signaling pathway	hsa04921	3.79 × 10^−5^	0.01319	
*MAPK1*	Oxytocin signaling pathway	hsa04921	3.79 × 10^−5^	0.01319	XPEHH
	GnRH signaling pathway	hsa04912	0.000534	0.050691	
	Thyroid hormone signaling pathway	hsa04919	0.001109	0.077681	
*RYR1*	Oxytocin signaling pathway	hsa04921	3.79 × 10^−5^	0.01319	XPEHH
*PDIA1*	Thyroid hormone synthesis	hsa04919	0.000367	0.045264	XPEHH
*CYP19A1*	Ovarian steroidogenesis	hsa04913	0.0020688	0.1079928	Fst/XPEHH

^1^ Corrected *p*-value here is a corrected *p*-value with FDR correction method Benjamini and Hochberg. ^2^ It represents the gene was detected by Fst or XPEHH test. For example, *ESR1* was detected by Fst test.

## Supplementary Materials

The following are available online at https://www.mdpi.com/2076-2615/10/9/1633/s1. Table S1: The results of Gene Ontology enrichment analyses, Table S2: The results of KEGG pathways enrichment analyses.
